# Awareness of a mesenteric mass as a common manifestation of ileal neuroendocrine tumor

**DOI:** 10.1186/s40792-020-00875-0

**Published:** 2020-05-24

**Authors:** Yosuke Kasai, Eric K. Nakakura

**Affiliations:** Department of Surgery, University of California, San Francisco, 1600 Divisadero Street, San Francisco, CA 94143-1932 USA

**Keywords:** Mesenteric mass, Ileal neuroendocrine tumor, Unknown primary

## Abstract

Omori et al. reported a case of multiple liver metastases originating from synchronous double cancer of “primary mesenteric neuroendocrine tumor” and rectal cancer. However, the “primary mesenteric neuroendocrine tumor” might be a misrecognition of mesenteric metastasis from ileal neuroendocrine tumor. Ileal neuroendocrine tumor is extremely rare in Japan. Herein, we aim to describe the characteristics of ileal neuroendocrine tumor and mesenteric mass as its common manifestation in reference to their reported case.

To the Editor,

Small intestine is the second common primary site of neuroendocrine tumors (NETs) in the USA [1], whereas the incidence of midgut NET is far less than those of pancreatic, foregut, and hindgut NETs in Japan [2]. Ileal NET (i-NET) accounts for most of the small intestinal NETs [3]. i-NETs are considered to originate from enterochromaffin cells and retain their capability to secrete biogenic amines and peptides, including serotonin [4]. These mediators activate cancer-associated fibroblast in the involved mesenteric lymph node, which results in the desmoplastic reaction of the mesentery and forms a large mesenteric mass (LMM) [4]. The latest American Joint Committee on Cancer TNM staging defined an LMM > 2 cm as N2 [5]. We recently showed that an LMM > 2 cm was present in 66 of 106 surgical cases (62%) with i-NET. Interestingly, the presence of LMM was not associated with liver metastasis or the extent of liver involvement, the strongest prognostic factor for i-NET. Moreover, the World Health Organization grade (G1 vs G2) was inversely correlated with LMM. LMM was independently associated with unfavorable prognosis (5-year survival rates of 64.8% and 92.9% for patients with and without LMM > 2 cm, respectively) [6].

Omori et al. reported a case of multiple liver metastases originating from synchronous double cancer of “primary mesenteric NET” and rectal cancer [7]. Although their evidence of “primary mesenteric NET” would be the absence of other primary foci on computed tomography and ^18^F-fludeoxyglucose positron emission tomography (^18^FDG-PET), ^18^FDG-PET is not sensitive for detecting low-grade primary gastrointestinal NETs [8]. As described above, a mesenteric mass is a common manifestation of i-NET, and their case might actually have a primary tumor in the ileum given the typical appearance of the mesenteric mass. Due to the small size and multifocality, consensus guidelines of the North American Neuroendocrine Tumor Society specifically recommends a “careful palpation” of the entire small bowel to detect primary i-NETs on surgical exploration [9]. In our previous study, 13 out of 15 patients with occult NET with liver metastasis were confirmed to have i-NETs by palpation [10]. ^68^Ga DOTA TOC/DOTA TATE-PET are highly sensitive for detecting NETs, but Norlén et al. showed that these modalities failed to detect 52% of primary small intestinal NETs that were detected by palpation on laparotomy, and concluded that palpation remained crucial regardless of the evolution of imaging modalities [11]. A question should be raised whether Omori et al. had done such careful palpation to seek for possible primary i-NET. Given the unfavorable prognosis of primary i-NET with LMM, the authors should have treated the mesenteric mass and possible primary i-NET, if present, as well as the rectal cancer.

In conclusion, although i-NET is rare in Japan, surgeons should be aware of a mesenteric mass as a common manifestation of i-NET not to miss the genuine primary tumor and to plan an appropriate therapeutic strategy based on the primary origin and the extent of tumor spread.

## Data Availability

Not applicable

## Abbreviations

^18^FDG-PET: ^18^F-fludeoxyglucose positron emission tomography
i-NET: Ileal neuroendocrine tumor
LMM: Large mesenteric mass
NET: Neuroendocrine tumor

## References

[1] Dasari A, Shen C, Halperin D, Zhao B, Zhou S, Xu Y (2017). Trends in the incidence, prevalence, and survival outcomes in patients with neuroendocrine tumors in the United States. JAMA Oncol..

[2] Ito T, Igarashi H, Nakamura K, Sasano H, Okusaka T, Takano K (2015). Epidemiological trends of pancreatic and gastrointestinal neuroendocrine tumors in Japan: a nationwide survey analysis. J Gastroenterol..

[3] Kim MK, Warner RR, Ward SC, Harpaz N, Roayaie S, Schwartz ME (2015). Prognostic significance of lymph node metastases in small intestinal neuroendocrine tumors. Neuroendocrinology..

[4] Blažević A, Hofland J, Hofland LJ, Feelders RA, de Herder WW (2018). Small intestinal neuroendocrine tumours and fibrosis: an entangled conundrum. Endocr Relat Cancer..

[5] Woltering EA, Bergsland EK, Beyer DT, O’Dorisio TM, Rindi G, Klimstra DS, et al. Neuroendocrine tumors of the jejunum and ileum. In: Amin MB, Edge S, Greene F, Byrd DR, Brookland RK, Washington MK, et al., editors. AJCC Cancer Staging Manual. 8th ed. New York: Springer; 2017. p. 375–87.

[6] Kasai Y, Mahuron K, Hirose K, Corvera CU, Kim GE, Hope TA (2019). Prognostic impact of a large mesenteric mass > 2 cm in ileal neuroendocrine tumors. J Surg Oncol..

[7] Omori S, Harada N, Toshima T, Takeishi K, Itoh S, Ikegami T (2020). Multiple liver metastases originating from synchronous double cancer of neuroendocrine tumor and rectal cancer: a case report. Surg Case Rep..

[8] Squires MH, Volkan Adsay N, Schuster DM, Russell MC, Cardona K, Delman KA (2015). Octreoscan versus FDG-PET for neuroendocrine tumor staging: a biological approach. Ann Surg Oncol..

[9] Howe JR, Cardona K, Fraker DL, Kebebew E, Untch BR, Wang YZ (2017). The surgical management of small bowel neuroendocrine tumors: consensus guidelines of the North American Neuroendocrine Tumor Society. Pancreas..

[10] Wang SC, Parekh JR, Zuraek MB, Venook AP, Bergsland EK, Warren RS (2010). Identification of unknown primary tumors in patients with neuroendocrine liver metastases. Arch Surg..

[11] Norlén O, Montan H, Hellman P, Stålberg P, Sundin A (2018). Preoperative ^68^Ga-DOTA-Somatostatin analog-PET/CT hybrid imaging increases detection rate of intra-abdominal small intestinal neuroendocrine tumor lesions. World J Surg..

